# A Combined Analysis of 48 Type 2 Diabetes Genetic Risk Variants Shows No Discriminative Value to Predict Time to First Prescription of a Glucose Lowering Drug in Danish Patients with Screen Detected Type 2 Diabetes

**DOI:** 10.1371/journal.pone.0104837

**Published:** 2014-08-26

**Authors:** Malene Hornbak, Kristine Højgaard Allin, Majken Linnemann Jensen, Cathrine Juel Lau, Daniel Witte, Marit Eika Jørgensen, Annelli Sandbæk, Torsten Lauritzen, Åsa Andersson, Oluf Pedersen, Torben Hansen

**Affiliations:** 1 The Novo Nordisk Foundation Center for Basic Metabolic Research, Faculty of Health and Medical Sciences, University of Copenhagen, Copenhagen, Denmark; 2 School of Pharmaceutical Sciences, Faculty of Health and Medical Sciences, University of Copenhagen, Copenhagen, Denmark; 3 Steno Diabetes Center A/S, Gentofte, Denmark; 4 Section for Social and Clinical Pharmacy, Department of Pharmacy, Faculty of Health and Medical Sciences, University of Copenhagen, Copenhagen, Denmark; 5 Research Centre for Prevention and Health, Capital Region of Denmark, Glostrup Hospital, Glostrup, Denmark; 6 Public Research Centre for Health, Centre for Health Studies, Strassen, Luxembourg; 7 Department of Public Health, Section of General Practice Medicine, Aarhus University, Aarhus, Denmark; 8 Institute of Biomedical Science, Faculty of Health and Medical Sciences, University of Copenhagen, Copenhagen, Denmark; 9 Faculty of Health Sciences, University of Aarhus, Aarhus, Denmark; 10 Section of Molecular Diabetes & Metabolism, Institute of Clinical Research & Institute of Molecular Medicine, Faculty of Health Sciences, University of Southern Denmark, Odense, Denmark; University of Catanzaro Magna Graecia, Italy

## Abstract

**Objective:**

To investigate the genetic influence of 48 type 2 diabetes susceptibility variants on disease progression measured as risk of early prescription redemption of glucose lowering drugs in screen-detected patients with type 2 diabetes.

**Methods:**

We studied type 2 diabetes progression in 1,480 patients with screen-detected type 2 diabetes from the ADDITION-Denmark study using information of redeemed prescriptions from the Register of Medicinal Products Statistics from 2001–2009 in Denmark. Patients were cluster randomized by general practitioners, who were randomized to treat type 2 diabetes according to either a conventional or a multifactorial intensive treatment algorithm. We investigated the genetic influence on diabetes progression by constructing a genetic risk score (GRS) of all 48 validated type 2 diabetes susceptibility variants, a GRS of 11 variants linked to β-cell function and a GRS of 3 variants linked to insulin sensitivity and assessed the association between number of risk alleles and time from diagnosis until first redeemed prescription of either any glucose lowering drug or an insulin drug.

**Results:**

The GRS linked to insulin sensitivity only nominally increased the risk of an early prescription redemption with an insulin drug by 39% (HR [95% C.I.] = 1.39 [1.09–1.77], p = 0.009] in patients randomized to the intensive treatment group. Furthermore, the strongest univariate predictors of diabetes progression for the intensive treatment group (measured as time to first insulin) were younger age (HR [95% C.I.] = 0.96 [0.93–0.99]), increased BMI (1.05 [1.01–1.09]), increased HbA1c (1.50 [1.36–.66]), increased TG (1.24 [1.11–1.39]) and reduced fasting serum HDL (0.37 [0.17–0.80]) at baseline. Similar results were obtained for the conventional treatment group.

**Conclusion:**

Higher levels of HbA1c, fasting circulating levels of triglyceride, lower HDL, larger BMI and younger age are significant determinants of early pharmacological intervention in type 2 diabetes. However, known common type 2 diabetes-associated gene variants do not appear to significantly affect disease progression.

## Introduction

The progression of type 2 diabetes (T2D) is linked to a progressive decline in pancreatic beta cell function with a parallel deterioration of glycemic control. The associated T2D pre-diabetic traits are highly versatile and complex, indicative of large disease heterogeneity and patients often progress at different rates. Despite multi-targeted treatment including life-style intervention and poly-pharmaceutical intervention, a high proportion of patients with T2D are not achieving sufficient target glycemic levels and often have HbA1c values well above those recommended by the American Diabetes Association [1] and the American Association of Clinical Endocrinologists [2]–[6]. A possible contributor is the continuous decline in β-cell function despite the immediate improvement following life-style intervention and oral glucose lowering drugs, and most patients will eventually require insulin therapy [7]–[10]. Hence, a large proportion of patients with T2D are at increased risk of microvascular and macrovascular complications and premature death despite the increased focus on early multi-targeted medical intervention.

Identifying biomarkers which can predict the progression rate of T2D as well as the response to treatment may have a beneficial impact on the prognosis of T2D. Several clinical risk markers (e.g. a family history of diabetes, body mass index (BMI), age and clinical features of the metabolic syndrome) are used today as indicative measures of progression from normoglycemic to hyperglycemic states [11], [12]. In addition, the extensive search into the genetic etiology of T2D has revealed >60 genetic variants which at a genome-wide significance level are associated with the disease [13]–[15]. Nonetheless, they only account for 5.7% of the variance in disease susceptibility [15] and the conventional clinical markers are still superior to genetic markers in predicting the diagnosis of type 2 diabetes [16]–[18]. Identifying genetic markers which influence the progressive loss of beta cell function and hence the increasing need for insulin treatment may increase our understanding and knowledge underlying the progressive and uncontrolled hyperglycemia despite medical intervention.

In the present study, the participants have been diagnosed with T2D through a screening program and followed by randomization to intensive care vs. standard care for five years. Hence, the study period involves the early stages of disease progression. Therefore, we first set out to investigate the influence of validated diabetes-associated gene variants on diabetes progression measured as an increased risk of early redemption of a glucose lowering drug prescription after diagnosis and secondly the increased risk of early redemption of an insulin drug prescription. Because the progressive decline in glucose regulation primarily reflects continuous deterioration of β-cell function we suspect that the genetic influence from particularly β-cell associating variants will have the largest impact. Hence we also investigated the genetic influence on disease progression stratified on genetic variants associating primarily with either β-cell or insulin sensitivity.

## Methods

### Participants

**Figure 1:**
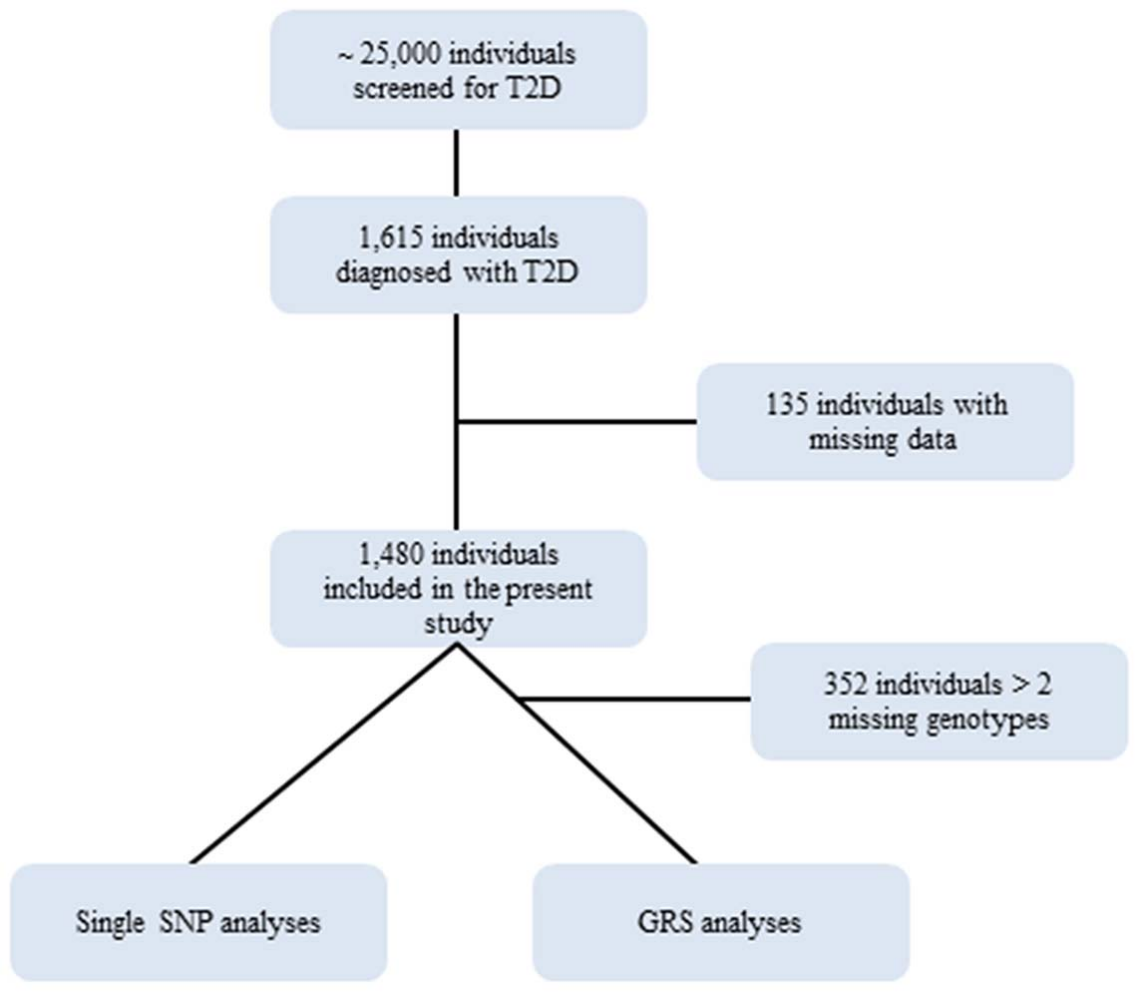
Flowchart over the number of participants in the present study. *GRS genetic risk score; SNP single nucleotide polymorphism; T2D type 2 diabetes.*

The Anglo-Danish-Dutch Study of Intensive Treatment in People With Screen-Detected Diabetes in Primary Care (ADDITION) study is a population-based screening and intervention study which was initiated in 2001 in the UK, The Netherlands and in Denmark. In Denmark the study was managed by the Department of Public Health, Section of General Practice Medicine at Aarhus University (ClinicalTrials.gov ID NCT00237548) and the study design has been described in detail elsewhere [19]. In short, individuals at high risk of diabetes, but without known diabetes, were initially identified using a self-administered questionnaire (based on age, sex, gestational diabetes, family history of diabetes, known hypertension, BMI and physical activity) [20]. From 2001–2006 approximately 160,000 individuals received a mailed invitation with the diabetes risk score questionnaire and ∼25,000 individuals with a high risk score (≥5) were invited to their general practitioner for measurements of random blood glucose and HbA1c [21], [22]. Individuals proceeded to a fasting blood glucose measurement and subsequently an oral glucose tolerance test (OGTT) if the fasting blood glucose was 5.6–6.1 or HbA1c≥5.8%. Patients with T2D were diagnosed by two independent diabetic plasma glucose values at baseline investigation according to WHO criteria [23]. Patients classified with T2D were subsequently invited to participate in an intervention study to investigate the effect of multifactorial intensive treatment over conventional treatment for T2D on cardiovascular mortality and morbidity with a follow-up of 5 years [19]. The two treatment groups included a conventional therapy group where patients were treated according to national recommendations for the management of T2D [24] and prevention of cardiovascular disease [25] and an intensive multifactorial treatment group where patients received both lifestyle advice and received guideline-driven multi-targeted management of blood glucose levels, blood pressure and cholesterol levels according to an intensive treatment algorithm [19]. By 2009 a total of 1,615 patients had been diagnosed with screen-detected T2D. However, 135 individuals were missing data on either baseline values or genotype information in the present study, leaving a total of 1,480 patients. The mean (SD) follow up time was 5.7 (3.0) years. A flowchart of the present study is shown in figure 1.

The study was in accordance with the Declaration of Helsinki revised in 1996 and approved by the Scientific Ethics Committee of Aarhus (# 20000183). All participants were of Danish nationality, and informed written consent was obtained from all patients before participation.

### Biochemical measurements and anthropometrics

Blood glucose was measured on capillary whole blood using a HemoCue B-glucose analyzer based on the glucose dehydrogenase reaction (HemoCue AB, Ängelholm, Sweden). For diagnosis, two capillary blood samples were taken and the average of the two results was used in order to minimize measurement error based on variation with HemoCue [26]. After an overnight fast, where the persons were instructed not to eat, drink or smoke later than 11pm the evening before, fasting venous blood glucose measurements and a 75-g OGTT were performed. HbA1c was analyzed using liquid chromatography on a Tosoh machine (TOSOH A1c 2.2; OSOH/Eurogenetics, Germany; normal range 4.2–6.3%). Fasting serum samples were analyzed for cholesterol, high-density lipoprotein (HDL) and Triglyceride (TG) using standard enzymatic methods. Low-density lipoprotein (LDL) was calculated using the Friedewald formula [27]. Height and weight were measured wearing light clothing and no shoes, and BMI was calculated as weight divided by the square of the height (kg/m^2^). Waist circumference was measured in standing position midway between the lowest rib and the iliac crest.

### Drug information and outcomes

Information on drug prescriptions was collected from the Register of Medicinal Product Statistics from the Danish Medicines Agency [28]. Since 1994 Danish pharmacies have collected information on every redeemed prescription electronically. In Denmark, medication for diabetes is only dispensed on doctor's prescription and can only be purchased from pharmacies. Each participant's cumulative medication profile was investigated from 2000–2010 which included information on drug classification under the anatomical therapeutic classification system (ATC) [29], pack size, strength of dose, date of purchase and amount of packages. As the participants were diagnosed with T2D through a screening program it appears reasonable to assume that the level of glycemic deterioration in the majority of the participants would be relatively low and that quite a few individuals would be able to be intervened purely on diet and physical exercise. Hence, diabetes progression was measured as time to first redeemed prescription of either any glucose-lowering drug (all drugs with ATC-code A10) or time to first redeemed prescription of an insulin drug (all drugs with ATC-code A10A). These two outcomes were defined as the period from diagnosis to the first appearance of a prescription. Participants with no prescriptions were right censored either in the event of death or reaching the end of follow-up. The final follow-up date was July 1^st^ 2009.

### SNP selection and genotyping

We selected SNPs previously shown to associate genome-wide significantly (p<5*10^−8^) with T2D in Caucasians based on the latest DIAGRAM publication [15]. Variants found to associate only with Asian populations were not included. Neither was *DUSP9* due to its location on the X-chromosome leaving 48 SNPs which were included in the analyses. 22 SNPs were genotyped by a custom-designed Illumina iSelect array (Illumina, San Diego, CA, USA) and 11 SNPs were genotyped by HumanExome Beadchip v1.0 array (Illumina, San Diego, CA, USA). For both sets of chip genotypings we removed closely related individuals, individuals with an extreme inbreeding coefficient, individuals with a low genotype call rate, individuals with mislabeled sex and individuals with a high discordance rate to previously genotyped SNPs. 15 SNPs were not present on either array and did not have perfect proxies and these were genotyped by a KASP genotyping assay (LGC Genomics (formerly KBioscience), Hoddeson, UK). Genotyping quality for each SNP was assessed by the success rate >96%, error rates <0.5% and the presence of Hardy-Weinberg equilibrium (p>0.01). 92% of the ADDITION participants fulfilled the quality criteria leaving a total of 1,480 individuals for the analyses. See table S1 for an overview of the 48 genotyped variants.

### Genetic risk score

We constructed a simple genetic risk score (GRS_Total) by summing up the number of risk alleles of all 48 variants for each individual assuming an additive effect of each allele. Individuals with more than 2 missing genotypes were excluded (n = 352) (table S2 depicts an overview of baseline characteristics of the group of individuals with more than two missing genotypes and the group of individuals with a maximum of two missing genotypes). Genotypes were imputed by assigning the most common genotype in ADDTION for the missing variant for individuals with 1 (n = 190) or 2 (n = 42) missing genotypes. The mean GRS was 50.2 (min-max: 36–65) risk alleles. Secondly, the genetic variants under study were grouped into two categories, inspired by [13], [30]; genetic variants affecting insulin secretion (termed GRS_beta: *TCF7L2*, *KCNQ1*, *MTNR1B*, *THADA*, *SLC30A8*, *CDKAL1*, *IGF2BP2*, *CENTD2*, *CDC123/CAMK1D* and *HNF1B*) and genetic variants affecting insulin sensitivity (termed GRS_SI: *FTO*, *PPARG* and *KLF14*) (table S1). Furthermore, we created a weighted GRS, as previously described [31], to evaluate possible differences between a simple and a weighted GRS. The weighted GRS was created by weighting each risk allele with the effect size (the natural log of the odds ratios) (See, table S1) for risk of T2D reported by the largest meta-analyses performed [15] and as previously done by [32]. No difference in results between the simple and the weighted GRS was observed and only results from the simple GRS are presented.

### Statistical analysis

The statistical analyses were performed using RGui version 3.0.1 ((http://www.r-project.org)) and SAS statistical software (version 9.2, SAS Institute Inc., Cary, USA). The analyses included all treatment naïve (e.g. no prior treatment of glucose lowering drugs) screen-detected patients with T2D in ADDITION-Denmark who entered the study program. The genetic influence on length of time between diabetes diagnosis to either 1) the first redeemed prescription of a glucose-lowering drug (termed 1^st^ drug for the remainder of the article) or 2) the first redemption of an insulin prescription (termed 1^st^ insulin for the remainder of the article) was investigated applying Cox proportional hazards regression analysis, adjusted for sex, age, BMI, Hba1c, LDL, HDL, TG, and smoking at study entry and general practitioner practice for each intervention group. The genetic impact was investigated primarily as the effect of all T2D susceptibility variants, calculated as three different GRS on disease progression in 1,128 individuals. To investigate the effect of each explanatory variable on disease progression we further performed univariate analyses in 1,128 individuals. Secondary analyses involved the influence of individual susceptibility genetic variants on disease progression in 1,480 individuals (figure 1). The assumption of proportional hazards was tested by the cox.zph function in R including all the explanatory variables in the model. No major violations were detected. To correct for multiple testing when performing single SNP analyses, Bonferroni correction was applied and a p<0.001 (0.05/48; number of SNPs under investigation) was considered significant. In the models including the GRS a Bonferroni corrected p<0.008 (0.05/6 analyses) was considered significant.

## Results

Baseline characteristics and summary statistics for patients in the intensive treatment group and the conventional treatment group are shown in table 1. Practices randomized to education in intensive treatment identified more patients with screen detected diabetes (n = 664) than practices randomized to conventional care (n = 464). Baseline characteristics were similar in the two groups with regards to median age (∼60.5 years), mean BMI (∼30.9 kg/m^2^), median HbA1c (∼6.4%), mean LDL (∼3.4 mmol/L), mean HDL (∼1.4 mmol/L) and median TG (∼1.6 mmol/L). However, the percentage of smokers were somewhat higher in the conventional group (37%) compared to the intensive group (32%) as was median time to 1^st^ redeemed glucose lowering drug (∼3.9 years in the conventional group versus ∼2.5 years in the intensive group). Median time to 1^st^ redeemed insulin prescription was similar in the two groups (∼6 years). Finally, the percentage of individuals who redeemed a glucose-lowering drug prescription was higher in the intensive group (∼67%) than in the conventional group (55%).

**Table 1:** Baseline characteristics and drug exposure for study participants.

	Intensive group	Conventional group
**N (men: women)**	664 (391∶273)	464 (264∶200)
**Age, years, median (IQR)**	60.6 (55.7–65.0)	60.3 (55.6–65.6)
**BMI, kg/m^2^, mean (SD)**	30.8 (5.4)	31.0 (5.4)
**HbA1c %, median (IQR)**	6.3 (6.0–7.0)	6.4 (6.0–6.9)
**LDL mmol/L, mean (SD)**	3.4 (1.0)	3.4 (1.0)
**HDL mmol/L, mean (SD)**	1.4 (0.4)	1.4 (0.4)
**Triglycerides mmol/L, median (IQR)**	1.6 (1.1–2.2)	1.6 (1.1–2.4)
**Number of smokers**	208 (32%)	169 (37%)
**Follow up time in years, median (IQR)**	6.1 (4.9–6.8)	6.0 (5.0–6.7)
**Time to 1^st^ redeemed glucose-lowering drug prescription in years, median (IQR)**	2.5 (0.3–5.6)	3.9 (1.9–6.1)
**Time to 1^st^ redeemed insulin prescription in years, median (IQR)**	5.9 (4.1–6.7)	6.0 (4.5–6.1)
**# of individuals with a glucose-lowering drug prescription (% of all participants)**	445 (67%)	256 (55%)
**Metformin**	405 (61.0%)	224 (48.0%)
**Sulphonylureas**	197 (29.7%)	103 (22.2%)
**Thiazolidinediones**	2 (0.3%)	5 (10.7%)
**α-glucosidase inhibitors**	0	1 (0.2%)
**GLP-1 analogs**	22 (3.3%)	15 (3.2%)
**DPP-IV inhibitors**	36 (5.4%)	28 (6.0%)
**Combination drugs**	30 (4.5%)	13 (2.8%)
**Insulin**	51 (7.7%)	26 (5.6%)

*BMI body mass index; HDL high density lipoprotein; IQR interquartile range; LDL low density lipoprotein; SD standard deviation. The glucose-lowering drugs contain the following ATC-codes: Metformin (A10BA02), Sulphonylureas (A10BB – 01, 03, 07, 09, 12), Thiazolidinediones (A10BG – 02, 03), α-glucosidase inhibitors (A10BF01), GLP-1 (A10BX – 04, 07), DPP-IVi (A10BH – 01, 02, 03), Combination drugs (A10BD – 03, 07, 08) and Insulin (A10AB – 01, 05, 06; A10AC01; A10AD01; A10AE04 and A10EE05).*

When we first examined the effect of the GRS including all T2D susceptibility variants (GRS_Total) on the risk of early redemption of a glucose lowering drug prescription in a multifactor-adjusted model neither time to 1^st^ drug nor time to 1^st^ insulin were affected by the GRS, 1^st^-drug-HR_intensive_ [95% C.I.] = 1.01 [0.99–1.04], p = 0.35 and 1^st^-drug-HR_conventional_ = 0.98 [0.95–1.02], p = 0.34 respectively and 1^st^-insulin-HR_intensive_ [95% C.I.] = 1.03 [0.97–1.09], p = 0.37 and 1^st^-insulin-HR_conventional_ = 0.88 [0.79–0.98], p = 0.02 respectively (table 2). Furthermore, we found no evidence that a genetic load of beta-cell associating variants have any influence on time from diagnosis until redemption of either 1^st^ drug or 1^st^ insulin prescription, 1^st^-drug-HR_intensive_ [95% C.I.] = 0.99 [0.94–1.04], p = 0.65 and 1^st^-drug-HR_conventional_ = 0.96 [0.90–1.03], p = 0.23 respectively and 1^st^-insulin-HR_intensive_ [95% C.I.] = 0.99 [0.87–1.12], p = 0.84 and 1^st^-insulin-HR_conventional_ = 1.03 [0.87–1.22], p = 0.70 respectively (table 2). A nominal association was, however, found between GRS_SI and time to 1^st^ redemption of an insulin prescription in the intensive treatment group (1^st^-insulin-HR_intensive_ = 1.39 [1.09–1.77], p = 0.009), suggesting that each unit of the GRS_SI score was associated with a 39% greater probability of redeeming an insulin prescription closer to time of diagnosis which can be translated into a faster progression to insulin treatment. Although non-significant, the insulin sensitivity GRS was associated with a 12% decreased risk of early insulin prescription redemption in the conventional treatment group.

**Table 2:** Adjusted Cox proportional hazards between genetic risk score and risk of early medical intervention.

		Time to 1^st^ drug			Time to 1^st^ insulin		
		HR [95% C.I.]	*P*-value	# of events	HR [95% C.I.]	*P*-value	# of events
**GRS_Total**	Intensive group* (N = 664)	[0.99–1.04	0.35	345	1.03 [0.97–1.09]	0.37	55
	Conventional group* (N = 464)	0.98 [0.95–1.02]	0.34	204	0.88 [0.79–0.98]	0.02	29
**GRS_beta**	Intensive group* (N = 664)	0.99 [0.94–1.04]	0.65	345	0.99 [0.87–1.12]	0.84	55
	Conventional group* (N = 464)	0.96 [0.90–1.03]	0.23	204	1.03 [0.87–1.22]	0.70	29
**GRS_SI**	Intensive group* (N = 664)	1.07 [0.97–1.17]	0.18	345	1.39 [1.09–1.77]	0.009	55
	Conventional group* (N = 464)	0.96 [0.84–1.10]	0.60	204	0.88 [0.61–1.28]	0.51	29

*Adjusted for age, sex, BMI, HbA1c, intervention, GP practice, HDL, LDL, TG, and smoking status at baseline. p-value<0.008 is considered significant.

The full multivariate Cox proportional hazards model for GRS_Total is presented in table 3 and univariate predictors of disease progression are summarized in table 4 for both the intensive treatment group and the conventional treatment group. The strongest univariate predictors of diabetes progression for the intensive treatment group (measured as time to1^st^ insulin) were younger age (HR [95% C.I.] = 0.96 [0.93–0.99]), increased BMI (1.05 [1.01–1.09]), increased HbA1c (1.50 [1.36–1.66]), increased TG (1.24 [1.11–1.39]) and reduced fasting serum HDL (0.37 [0.17–0.80]) at baseline. Similar results were obtained for the conventional treatment group, though only increased HbA1c and TG at baseline were statistically significant predictors.

**Table 3:** Adjusted multivariate Cox proportional hazards model for diabetes progression measured as risk of early insulin prescription redemption in the intensive treatment group and the conventional treatment group.

Intensive group	HR [95% C.I.]	*P*-value	Conventional group	HR [95% C.I.]	*P*-value
**GRS_Total**	1.03 [0.97–1.09]	0.37	**GRS_Total**	0.88 [0.79–0.98]	0.02
**Age at baseline (yrs.)**	0.99 [0.95–1.03]	0.59	**Age at baseline (yrs.)**	0.99 [0.93–1.05]	0.70
**Sex (males vs. female)**	0.99 [0.55–1.80]	0.98	**Sex (males vs. female)**	1.16 [0.47–2.88]	0.74
**BMI (kg/m^2^)**	1.04 [0.99–1.08]	0.13	**BMI (kg/m^2^)**	0.99 [0.92–1.07]	0.85
**HbA1c (%)**	1.46 [1.30–1.63]	**8.63E10^−11^**	**HbA1c (%)**	1.74 [1.43–2.12]	**2.97E10^−8^**
**LDL (mmol/L)**	1.12 [0.87–1.45]	0.37	**LDL (mmol/L)**	0.73 [0.48–1.12]	0.15
**HDL (mmol/L)**	1.09 [0.42–2.81]	0.87	**HDL (mmol/L)**	0.12 [0.02–0.67]	0.02
**TG (mmol/L)**	1.31 [1.10–1.56]	**2.92E10^−3^**	**TG (mmol/L)**	0.93 [0.66–1.32]	0.70
**Smoking status (non-smokers vs. smokers)**	1.00 [0.54–1.84]	0.99	**Smoking status (non-smokers vs. smokers)**	0.92 [0.40–2.11]	0.84

**Table 4:** Univariate associations with diabetes progression measured as risk of early insulin prescription redemption in the intensive treatment group and the conventional treatment group.

Intensive group	HR [95% C.I.]	*P*-value	Conventional group	HR [95% C.I.]	*P*-value
**GRS_Total**	1.03 [0.97–1.08]	0.28	**GRS_Total**	0.93 [0.86–1.01]	0.10
**GRS_beta**	0.98 [0.87–1.10]	0.70	**GRS_beta**	1.01 [0.87–1.16]	0.93
**GRS_SI**	1.22 [0.99–1.51]	0.07	**GRS_SI**	0.98 [0.72–1.32]	0.88
**Age at baseline (yrs.)**	0.96 [0.93–0.99]	**0.01**	**Age at baseline (yrs.)**	0.99 [0.93–1.05]	0.70
**Sex (males vs. female)**	0.96 [0.60–1.55]	0.90	**Sex (males vs. female)**	1.36 [0.69–2.67]	0.38
**BMI (kg/m^2^)**	1.05 [1.01–1.09]	**0.02**	**BMI (kg/m^2^)**	1.03 [0.96–1.09]	0.43
**HbA1c (%)**	1.50 [1.36–1.66]	**9.99E10^−16^**	**HbA1c (%)**	1.52 [1.31–1.77]	**3.38E10^−8^**
**LDL (mmol/L)**	1.17 [0.91–1.50]	0.22	**LDL (mmol/L)**	0.85 [0.58–1.23]	0.38
**HDL (mmol/L)**	0.37 [0.17–0.80]	**0.01**	**HDL (mmol/L)**	0.14 [0.04–0.47]	**1.57E10^−3^**
**TG (mmol/L)**	1.24 [1.11–1.39]	**1.93E10^−4^**	**TG (mmol/L)**	1.07 [0.97–1.18]	0.17
**Smoking status (non-smokers vs. smokers)**	1.17 [0.71–1.92]	0.54	**Smoking status (non-smokers vs. smokers)**	1.36 [0.69–2.67]	0.38

Finally, we investigated the impact of single gene variants on disease progression (table S1). After Bonferroni correction, no single variants significantly associated with either progression to 1^st^ drug or 1^st^ insulin.

## Discussion

In the present study we have investigated the effect of known T2D susceptibility genetic variants on disease progression measured as early redemption of either a glucose lowering drug or an insulin drug prescription in screen-detected patients with T2D. Only the GRS linked to insulin sensitivity nominally increased the risk of early prescription redemption with an insulin drug in the intensive treatment group, whereas the two other GRS did not show an effect on disease progression in either intervention group. Consistent with the literature we show that increased HbA1c, BMI, TG, low HDL and younger age are independent predictors of disease progression [33]–[35].

If diabetes susceptibility variants affect disease progression we would expect a significant association between an increasing gene-load and early medical intervention. However, we were not able to detect any association between either the GRS_Total or GRS_beta scores and early prescription redemption. Besides the possibility that disease progression is not genetically influenced, possibly different unidentified genetic variants affect disease progression which has also been proposed by Zhou et al [33]. A future whole exome-wide or whole genome-wide association study followed by a region burden analysis may reveal putative novel gene variants which affect disease progression. Another explanation could reside in the selection of variants included in the GRS. Previous studies have included a variety of variants in their GRS-categorization and additional gene*gene interactions (e.g. additive, synergistic or opposing effects) will undoubtedly affect the overall gene load associated with risk of progression [36]. Correspondingly, a study by Iwata et al [30] investigated the association of 14 susceptibility alleles for T2D and use of insulin therapy in 724 T2D patients with a mean duration of diabetes of 13.6 years. They found a link between their beta-cell GRS and use of insulin and concluded that patients with a higher proportion of disease susceptibility variants related to β-cell function were associated with reduced basal insulin secretion over time which contributes to the need for insulin injections. We therefore tried to include different variants in our GRS_beta inspired by Iwata et al [37], by Zhou K et al [33], by Kahn et al [38] and by Rosengren et al [39]. However, none of the beta cell scores were associated with early prescription redemption in our study material (data not shown). Nonetheless, other factors will also affect the association, e.g. number of participants included in the study, duration of study and state of disease progression (newly screen-detected patients versus patients with known diabetes of variable length). Furthermore, it is hard to adjust for other factors which may influence disease progression, such as diet, physical activity and importantly adherence to medical therapy. Hence patients who are adherent to their oral glucose lowering treatment regiments may postpone their glycemic deterioration for a longer period of time and thereby also postpone their need for insulin treatment).

Several studies have demonstrated that the progressive decline in β-cell function begins several years prior to diagnosis and continues as disease progresses [9], [40]–[42]. The U.K. Prospective Diabetes Study (UKPDS) [43] showed that increasing hyperglycemia over time was associated with a decline of surrogate measures of β-cell function and was not associated with changes in insulin sensitivity (both assessed by HOMA approaches). Moreover, evidence suggests that loss of β-cell function in T2D patients is the major determinant of disease progression compared to insulin resistance which leads to initiation of oral glucose lowering polypharmacy and subsequent use of insulin. To our surprise, we only detected a nominal association between the GRS_SI and disease progression measured as early redemption of an insulin prescription in the intensive group which has not been seen previously. Moreover, the GRS_SI was associated with a 12% decreased risk of early insulin prescription redemption in the conventional group, though not significant. As table 1 indicates, a larger proportion of patients in the intensive treatment group have redeemed a glucose-lowering drug prescription and an insulin prescription compared to patients in the conventional treatment group (67% vs. 55% and 7.7% vs. 5.6% respectively) which is as expected and in line with the protocol guidelines. Consequentially, the more ‘aggressive’ pharmacological approach in the intensive group compared to the conventional group may drive the hazard ratios in opposite directions. Conclusively, the association between GRS_SI and disease progression is likely to be a spurious finding and will require further investigation.

Nevertheless, why do we not see an effect from the GRS_beta score when we know that these gene variants are significantly associated with beta cell function and that diabetes progression is linked to a progressive decline in beta cell function? Previous studies have shown that older T2D patients are more insulin resistant while a more aggressive decline in β-cell function predominates in younger patients [44]–[46]. In relation to this, our study participants, who have a median age of 60 and are in the early stages of diabetes progression, may represent a more insulin resistant phenotype in which the functional β-cell machinery is still able to compensate for diminishing insulin sensitivity. In the intensive therapy arm of the ADDITION study protocol the participants were recommended to have insulin if their HbA1c level remained above 7% after initial oral mono- or dual glucose lowering therapy or otherwise determined by the individual general practitioner [19]. In relation to this we cannot assume that the participants who have redeemed an insulin prescription actually have a deficient β-cell function. In fact, it could be the result of heterogeneous (non-consistent) prescription patterns among the participating general practitioners or non-compliance of the general practitioner to apply to study guidelines. Moreover, if our study participants represent a more insulin resistant phenotype they may simply be more susceptible to environmental elements (e.g. hypercaloric diet and sedentary lifestyle) which leads to hyperglycemia and hence HbA1c>7% which may precede β-cell dysfunction [42], [47]. Consequently, such potential scenario may explain why we only see a link between disease progression and the GRS_SI but not with the other GRSs.

Despite an immediate improvement in β-cell function with initiation of medical therapy, a progressive loss of β-cell function is still observed over time suggesting underlying pathophysiological mechanisms that are independent of the initial pharmacological effect on the β-cell [9], [48], [49]. This is substantiated by the increasing rise in HbA1c over time even in patients on medical intervention [50]. Furthermore, the progressive deterioration in glycemic control is often explained by gluco- and lipotoxicity which directly affect the β-cells and insulin sensitivity [42], [51], [52]. Hence, investigating the association between genetic loci linked with either HbA1c or gluco- and lipotoxic properties and time to redemption of 1^st^ drug and 1^st^ insulin prescription may also reveal links to diabetes progression. A statistically well-powered genome-wide association study may reveal such loci and possibly also new and previously unidentified variants.

Studying the genetic prediction of disease progression measured as the time between diabetes onset and time to 1^st^ redemption of a glucose-lowering drug prescription should be simple and give well-defined outcome measures. There are however several influential factors which may affect the results of such studies which need to be taken into account. In the present study, we have been privileged to follow individuals in the very early stages of diabetes progression for approximately 6 years and follow their prescription patterns through the Register of Medicinal Products Statistics of Denmark. This is a unique registry which makes it possible to perform pharmaco-epidemiological studies of high quality and accuracy [53]. Nevertheless, only 67% and 8%, respectively, of the patients in the intensive treatment group redeemed a prescription of glucose-lowering drugs and insulin (55% and 6% respectively in the conventional treatment group). These numbers could arise from physician bias where prescriber decisions are influenced by previous experience which may lead to non-adherence of the ADDITION treatment guidelines. If this is the case, the time to 1^st^ prescription redemption may depend more on the general practitioner than on the ADDITION treatment guidelines and hence may mask putative clinical and genetic predictors of disease progression (measured as time to 1^st^ redemption). Furthermore, since the participants have been screen-detected and therefore are in the early stages of diabetes, their ability to maintain glycemic control by diet and exercise alone is greater. A situation which may be influenced by increased health interest from those who accepted to participate in the study. Ringborg A et al (2010) studied factors influencing time from initiation of oral glucose lowering drugs to start of insulin use among ∼5,000 T2D patients and found the mean time to insulin use was 4.0+/−2.8 years (during 1994–2005) [34]. Clearly a longer study follow up would have allowed us to capture more individuals progressing to medical intervention. A longer study time together with a larger study population would undoubtedly increase the number of observed redeemed prescriptions and hence the statistical power of the analyses. Nevertheless, the confidence intervals for the various hazard ratios are generally narrow indicating a high specificity of the analyses, we have obtained a significant effect of known clinical risk factors in accordance with previous studies [35], [54] and our results conform with results from a similar study by Zhou K and co-workers who included >5,000 individuals in their study [33].

## Conclusions

In contrast to confirmed clinical biomarkers, known diabetogenic variants do not appear to significantly affect the time to redemption of 1^st^ drug or 1^st^ insulin prescription after disease onset. We suggest that other genetic biomarkers are involved in the process and therefore propose a genome wide association study and a gene burden analysis to pinpoint previously unidentified markers which may help expand our understanding of the biochemical mechanisms behind diabetes progression and why glycaemia persists to deteriorate despite medical treatment.

## Supporting Information

Table S1 **48 European type 2 diabetes SNPs genotyped in the ADDITION-DK cohort used for GRS construction and single biomarker analyses, N = 1,480.** ^a^ OR for risk of T2D used for weighting the GRS, as done in Andersson EA et al [32]; ^b^ for proxy SNP not in LD (rs<0.8) with corresponding SNP reported by in Andersson EA et al [32], the OR was obtained from another genome-wide association study; ^c^ SNP in linkage disequilibrium (r2>0.8) with the corresponding SNP reported by in Andersson EA et al [32]; ^d^ Alleles aligned to the forward strand of NCBI Build 37.5; ^e^ Risk allele according to NCBI Build 37.5; ^f^ RAF = risk allele frequency in the ADDITION-DK cohort; ^g^ Adjusted for sex, age, BMI, HbA1c, HDL, LDL, TG, and smoking at baseline and intervention group and GP practice; ^beta^ and ^SI^ denotes the categorization of genes included in the genetic risk score of either beta-cell or insulin sensitivity variants, respectively.
(DOCX)

Table S2 **Baseline characteristics for patients with more than 2 missing genotypes and for patients with a maximum of 2 missing genotypes.** *BMI body mass index; HDL high density lipoprotein; IQR interquartile range; LDL low density lipoprotein; SD standard deviation. The glucose-lowering drugs contain the following ATC-codes: Metformin (A10BA02), Sulphonylureas (A10BB – 01, 03, 07, 09, 12), Thiazolidinediones (A10BG – 02, 03), α-glucosidase inhibitors (A10BF01), GLP-1 (A10BX – 04, 07), DPP-IVi (A10BH – 01, 02, 03), Combination drugs (A10BD – 03, 07, 08) and Insulin (A10AB – 01, 05, 06; A10AC01; A10AD01; A10AE04 and A10EE05).*
(DOCX)
