# Supplementary material for: A Combined Analysis of 48 Type 2 Diabetes Genetic Risk Variants Shows No Discriminative Value to Predict Time to First Prescription of a Glucose Lowering Drug in Danish Patients with Screen Detected Type 2 Diabetes
Source: PLoS One. 2014 Aug 26;9(8):e104837. doi: 10.1371/journal.pone.0104837 (PMC4144838; doi:10.1371/journal.pone.0104837)
Supplement: Table S1 — 48 European type 2 diabetes SNPs genotyped in the ADDITION-DK cohort used for GRS construction and single biomarker analyses, N = 1,480. a OR for risk of T2D used for weighting the GRS, as done in Andersson EA et al [32]; b for proxy SNP not in LD (rs<0.8) with corresponding SNP reported by in Andersson EA et al [32], the OR was obtained from another genome-wide association study; c SNP in linkage disequilibrium (r2>0.8) with the corresponding SNP reported by in Andersson EA et al [32]; d Alleles aligned to the forward strand of NCBI Build 37.5; e Risk allele according to NCBI Build 37.5; f RAF = risk allele frequency in the ADDITION-DK cohort; g Adjusted for sex, age, BMI, HbA1c, HDL, LDL, TG, and smoking at baseline and intervention group and GP practice; beta and SI denotes the categorization of genes included in the genetic risk score of either beta-cell or insulin sensitivity variants, respectively. (DOCX) [file pone.0104837.s001.docx]

Table S1. 48 European type 2 diabetes SNPs genotyped in the ADDITION-DK cohort used for GRS construction and single biomarker analyses, N=1,480.

| **Gene** | **SNP** | **Chr** | **Genotyping** | **OR^a^** | **Alleles^d^ (risk^e^/other)** | **RAF^f^** | **HR^g^ [95% CI], time_to_first_drug** | ***P*** | **HR^g^ [95% CI], time_to_first_insulin** | ***P*** |
| --- | --- | --- | --- | --- | --- | --- | --- | --- | --- | --- |
| *TCF7L2^beta^* | rs7903146 | 10 | KASP genotyping | 1.39 | T/C | 0.32 | 1.01 [0.89-1.15] | 0.86 | 1.09 [0.77-1.48] | 0.61 |
| *KCNQ1^beta^* | rs2237895 | 11 | KASP genotyping | 1.24^b^ [1] | C/A | 0.45 | 0.89 [0.80-1.00] | 0.05 | 0.86 [0.62-1.12] | 0.37 |
|  | rs231362 | 11 | Illumina iSelect | 1.08 | G/A | 0.53 | 1.04 [0.93-1.16] | 0.51 | 1.03 [0.74-1.32] | 0.87 |
| *MTNR1B^beta^* | rs10830963 | 11 | Illumina iSelect | 1.10 | G/C | 0.31 | 1.00 [0.88-1.13] | 0.94 | 0.92 [0.64-1.28] | 0.64 |
| *THADA ^beta^* | rs7578597 | 2 | Illumina iSelect | 1.14 | T/C | 0.90 | 1.06 [0.89-1.29] | 0.55 | 1.00 [0.63-1.71] | 0.99 |
| *SLC30A8^beta^* | rs13266634 | 8 | Illumina iSelect | 1.14 | C/T | 0.70 | 0.95 [0.83-1.07] | 0.44 | 1.25 [0.89-1.71] | 0.19 |
| *CDKAL1^beta^* | rs10946398 | 6 | KASP genotyping | 1.12^b^ [2] | C/A | 0.36 | 1.04 [0.92-1.17] | 0.51 | 1.03 [0.74-1.35] | 0.87 |
| *IGF2BP2^beta^* | rs4402960 | 3 | Illumina iSelect | 1.13 | T/G | 0.33 | 0.98 [0.86-1.10] | 0.75 | 0.84 [0.61-1.18] | 0.32 |
| *CENTD2/ ARAP1^beta^* | rs1552224 | 11 | Illumina iSelect | 1.11 | A/C | 0.84 | 0.87 [0.75-1.01] | 0.08 | 0.73 [0.50-1.07] | 0.11 |
| *CDC123/ CAMK1D ^beta^* | rs12779790 | 10 | KASP genotyping | 1.07 | G/A | 0.21 | 1.15 [1.01-1.33] | 0.06 | 1.08 [0.72-1.41] | 0.68 |
| *HNF1B ^beta^* | rs7501939 | 17 | Illumina iSelect | 1.10 | C/T | 0.59 | 0.89 [0.78-0.98] | 0.02 | 1.11 [0.82-1.54] | 0.52 |
| *FTO ^SI^* | rs9939609 | 16 | KASP genotyping | 1.14 | A/T | 0.43 | 1.00 [0.90-1.13] | 0.97 | 1.21 [0.88-1.61] | 0.25 |
| *PPARG ^SI^* | rs1801282 | 3 | KASP genotyping | 1.13 | C/G | 0.87 | 1.10 [0.88-1.23] | 0.29 | 0.91 [0.67-1.60] | 0.68 |
| *KLF14^SI^* | rs972283 | 7 | Illumina iSelect | 1.04 | G/A | 0.50 | 1.09 [0.97-1.23] | 0.15 | 1.37 [0.91-1.66] | 0.05 |
| *CDKN2A/B* | rs10811661 | 9 | KASP genotyping | 1.18 | T/C | 0.86 | 0.93 [0.78-1.10] | 0.37 | 0.97 [0.63-1.48] | 0.91 |
| *HHEX* | rs1111875 | 10 | KASP genotyping | 1.11 | C/T | 0.59 | 1.05 [0.93-1.19] | 0.43 | 1.25 [0.89-1.71] | 0.18 |
| *KCNJ11* | rs5219 | 11 | KASP genotyping | 1.07 | T/C | 0.40 | 0.95 [0.84-1.06] | 0.43 | 0.67 [0.54-0.99] | 0.02 |
| *WFS1* | rs10010131 | 4 | Illumina iSelect | 1.10 | G/A | 0.58 | 0.90 [0.81-1.02] | 0.07 | 0.80 [0.06-1.11] | 0.16 |
| *JAZF1* | rs864745 | 7 | Illumina iSelect | 1.11 | T/C | 0.53 | 1.06 [0.94-1.19] | 0.37 | 1.33 [0.95-1.76] | 0.08 |
| *TSPAN8* | rs7961581 | 12 | Illumina iSelect | 1.06 | C/T | 0.28 | 1.04 [0.89-1.17] | 0.55 | 0.95 [0.68-1.36] | 0.78 |
| *GCK* | rs1799884 | 7 | KASP genotyping | 1.08 | A/G | 0.17 | 0.94 [0.82-1.10] | 0.42 | 0.86 [0.63-1.37] | 0.49 |
| *PROX1* | rs340874 | 1 | Illumina iSelect | 1.07 | C/T | 0.56 | 1.01 [0.92-1.15] | 0.83 | 1.06 [0.80-1.45] | 0.73 |
| *DGKB/ TMEM195* | rs2191349 | 7 | Illumina iSelect | 1.05 | T/G | 0.47 | 0.99 [0.91-1.14] | 0.92 | 1.59 [1.11-2.05] | 0.01 |
| *C2CD4* | rs7172432 | 15 | Illumina iSelect | 1.06 | A/G | 0.60 | 1.06 [0.96-1.20] | 0.33 | 0.86 [0.66-1.20] | 0.33 |
| *BCL11A* | rs243021 | 2 | Illumina iSelect | 1.07^c^ | A/G | 0.50 | 1.02 [0.90-1.12] | 0.78 | 0.87 [0.61-1.11] | 0.37 |
| *ADCY5* | rs11708067 | 3 | Illumina iSelect | 1.11 | A/G | 0.78 | 1.03 [0.89-1.17] | 0.64 | 0.83 [0.57-1.17] | 0.32 |
| *ANK1* | rs516946 | 8 | Exome chip | 1.09 | C/T | 0.80 | 0.97 [0.83-1.11] | 0.65 | 1.07 [0.73-1.60] | 0.75 |
| *BCAR1* | rs7202877 | 16 | Exome chip | 1.12 | T/G | 0.91 | 1.10 [0.87-1.34] | 0.41 | 1.24 [0.69-2.10] | 0.46 |
| *IRS1* | rs2943641 | 2 | Illumina iSelect | 1.19 | C/T | 0.65 | 1.03 [0.91-1.16] | 0.62 | 0.86 [0.66-1.25] | 0.35 |
| *ADAMTS9* | rs4607103 | 3 | Illumina iSelect | 1.08 | C/T | 0.78 | 1.09 [0.91-1.22] | 0.27 | 0.92 [0.64-1.39] | 0.69 |
| *GCKR* | rs780094 | 2 | KASP genotyping | 1.09 | C/T | 0.67 | 0.96 [0.84-1.06] | 0.55 | 1.02 [0.79-1.49] | 0.92 |
| *RBMS1* | rs7593730 | 2 | KASP genotyping | 1.04^c^ | A/G | 0.20 | 1.06 [0.89-1.18] | 0.43 | 0.89 [0.58-1.24] | 0.53 |
| *GRB14* | rs13389219 | 2 | Exome chip | 1.07 | C/T | 0.60 | 1.09 [0.97-1.23] | 0.17 | 1.26 [0.95-1.80] | 0.15 |
| *ANKRD55* | rs459193 | 5 | Exome chip | 1.08 | G/A | 0.75 | 0.95 [0.84-1.09] | 0.45 | 0.88 [0.65-1.27] | 0.46 |
| *HMGA2* | rs1531343 | 12 | Exome chip | 1.12 | C/G | 0.09 | 0.94 [0.80-1.22] | 0.61 | 0.74 [0.46-1.45] | 0.33 |
| *NOTCH2* | rs10923931 | 1 | KASP genotyping | 1.08 | T/G | 0.10 | 1.20 [0.99-1.41] | 0.06 | 1.30 [0.81-1.96] | 0.27 |
| *CHCHD9/ TLE4* | rs13292136 | 9 | KASP genotyping | 1.12 | C/T | 0.92 | 0.97 [0.78-1.17] | 0.80 | 0.72 [0.50-1.40] | 0.22 |
| *HNF1A* | rs7957197 | 12 | Illumina iSelect | 1.08 | T/A | 0.80 | 1.00 [0.90-1.19] | 0.95 | 0.83 [0.58-1.18] | 0.32 |
| *ZBED3* | rs4457053 | 5 | Illumina iSelect | 1.10 | G/A | 0.30 | 0.98 [0.88-1.13] | 0.76 | 0.91 [0.67-1.26] | 0.59 |
| *PRC1* | rs8042680 | 15 | KASP genotyping | 1.07 | G/A | 0.33 | 1.00 [0.87-1.12] | 0.99 | 1.03 [0.73-1.43] | 0.87 |
| *TP53INP1* | rs896854 | 8 | Illumina iSelect | 1.05 | T/C | 0.44 | 1.03 [0.91-1.14] | 0.63 | 1.11 [0.76-1.36] | 0.52 |
| *ZFAND6* | rs11634397 | 15 | Illumina iSelect | 1.05 | G/A | 0.68 | 0.92 [0.80-1.04] | 0.16 | 0.90 [0.67-1.22] | 0.52 |
| *TLE1* | rs2796441 | 9 | Exome chip | 1.07 | G/A | 0.60 | 1.13 [1.00-1.27] | 0.05 | 0.83 [0.61-1.13] | 0.24 |
| *ZMIZ1* | rs12571751 | 10 | Exome chip | 1.08 | A/G | 0.53 | 1.08 [0.93-1.17] | 0.20 | 1.19 [0.87-1.60] | 0.27 |
| *KLHDC5* | rs10842994 | 12 | Exome chip | 1.10 | C/T | 0.81 | 1.00 [0.86-1.16] | 1.00 | 0.97 [0.71-1.60] | 0.87 |
| *HMG20A* | rs7177055 | 15 | Exome chip | 1.08 | A/G | 0.73 | 0.91 [0.75-1.15] | 0.41 | 0.80 [0.48-1.45] | 0.46 |
| *MC4R* | rs12970134 | 18 | Exome chip | 1.08 | A/G | 0.29 | 0.98 [0.84-1.09] | 0.77 | 0.79 [0.54-1.05] | 0.17 |
| *CILP2* | rs10401969 | 19 | Exome chip | 1.13 | C/T | 0.09 | 0.91 [0.77-1.18] | 0.39 | 0.83 [0.52-1.72] | 0.55 |

^a^ OR for risk of T2D used for weighting the GRS, as done in Andersson EA et al [3]; ^b^ for proxy SNP not in LD (rs<0.8) with corresponding SNP reported by in Andersson EA et al [3], the OR was obtained from another genome-wide association study; ^c^ SNP in linkage disequilibrium (r2>0.8) with the corresponding SNP reported by in Andersson EA et al [3]; ^d^ Alleles aligned to the forward strand of NCBI Build 37.5; ^e^ Risk allele according to NCBI Build 37.5; ^f^ RAF= risk allele frequency in the ADDITION-DK cohort; ^g^ Adjusted for sex, age, BMI, HbA1c, HDL, LDL, TG, and smoking at baseline and intervention group and GP practice; ^beta^ and ^SI^ denotes the categorization of genes included in the genetic risk score of either beta-cell or insulin sensitivity variants, respectively.

Reference List

1. Unoki H, Takahashi A, Kawaguchi T, et al (2008) SNPs in KCNQ1 are associated with susceptibility to type 2 diabetes in East Asian and European populations. Nat.Genet. 40: 1098-1102

2. Zeggini E, Scott LJ, Saxena R, et al (2008) Meta-analysis of genome-wide association data and large-scale replication identifies additional susceptibility loci for type 2 diabetes. Nat.Genet. 40: 638-645

3. Andersson EA, Allin KH, Sandholt CH, et al (2013) Genetic risk score of 46 type 2 diabetes risk variants associates with changes in plasma glucose and estimates of pancreatic beta-cell function over 5 years of follow-up. Diabetes 62: 3610-3617
