# Supplementary material for: A Combined Analysis of 48 Type 2 Diabetes Genetic Risk Variants Shows No Discriminative Value to Predict Time to First Prescription of a Glucose Lowering Drug in Danish Patients with Screen Detected Type 2 Diabetes
Source: PLoS One. 2014 Aug 26;9(8):e104837. doi: 10.1371/journal.pone.0104837 (PMC4144838; doi:10.1371/journal.pone.0104837)
Supplement: Table S2 — Baseline characteristics for patients with more than 2 missing genotypes and for patients with a maximum of 2 missing genotypes. BMI body mass index; HDL high density lipoprotein; IQR interquartile range; LDL low density lipoprotein; SD standard deviation. The glucose-lowering drugs contain the following ATC-codes: Metformin (A10BA02), Sulphonylureas (A10BB – 01, 03, 07, 09, 12), Thiazolidinediones (A10BG – 02, 03), α-glucosidase inhibitors (A10BF01), GLP-1 (A10BX – 04, 07), DPP-IVi (A10BH – 01, 02, 03), Combination drugs (A10BD – 03, 07, 08) and Insulin (A10AB – 01, 05, 06; A10AC01; A10AD01; A10AE04 and A10EE05). (DOCX) [file pone.0104837.s002.docx]

Table S2. Baseline characteristics for patients with more than 2 missing genotypes and for patients with a maximum of 2 missing genotypes.

|  | ≤ 2 missing genotypes, N = 1,128 | > 2 missing genotypes, N = 352 |
| --- | --- | --- |
| N (men: women) | 1,128 (655:473) | 352 (191:161) |
| Intervention group (intensive : conventional) | 664:464 | 213:139 |
| Age, years, median (IQR) | 60.5 (55.6-65.2) | 60.8 (54.4-65.4) |
| BMI, kg/m^2^, mean (SD) | 30.9 (5.4) | 30.4 (5.5) |
| HbA1c %, median (IQR) | 6.4 (6.0-7.0) | 6.3 (6.0-6.9) |
| LDL mmol/L, mean (SD) | 3.4 (1) | 3.4 (1) |
| HDL mmol/L, mean (SD) | 1.4 (0.4) | 1.4 (0.4) |
| Triglycerides mmol/L, median (IQR) | 1.6 (1.1-2.3) | 1.6 (1.1-2.3) |
| Number of smokers | 377 (33%) | 120 (34%) |
| Follow up time in years, median (IQR) | 6.0 (4.6-6.7) | 5.8 (3.8-6.5) |
| Time to 1^st^ redeemed glucose-lowering drug prescription in years, median (IQR) | 3.4 (0.7-5.9) | 3.1 (0.6-5.3) |
| Time to 1^st^ redeemed insulin prescription in years, median (IQR) | 6.0 (4.2-6.6) | 5.7 (3.7-6.4) |
| # of individuals with a glucose-lowering drug prescription (% of all participants) | 726 (64%) | 230 (65%) |
| Metformin | 647 (57%) | 204 (58%) |
| Sulphonylureas | 300 (27%) | 90 (26%) |
| Thiazolidinediones | 7 (0.6%) | 2 (0.6%) |
| α-glucosidase inhibitors | 1 (0.0009%) | 1 (0.3%) |
| GLP-1 analogs | 37 (3%) | 10 (3%) |
| DPP-IV inhibitors | 64 (6%) | 16 (5%) |
| Combination drugs | 43 (4%) | 18 (5%) |
| Insulin | 77 (7%) | 23 (7%) |

*BMI body mass index; HDL high density lipoprotein; IQR interquartile range; LDL low density lipoprotein; SD standard deviation. The glucose-lowering drugs contain the following ATC-codes: Metformin (A10BA02), Sulphonylureas (A10BB – 01, 03, 07, 09, 12), Thiazolidinediones (A10BG – 02, 03), α-glucosidase inhibitors (A10BF01), GLP-1 (A10BX – 04, 07), DPP-IVi (A10BH – 01, 02, 03), Combination drugs (A10BD – 03, 07, 08) and Insulin (A10AB – 01, 05, 06; A10AC01; A10AD01; A10AE04 and A10EE05).*
